# Supplementary material for: Attention amplifies neural representations of changes in sensory input at the expense of perceptual accuracy
Source: Nat Commun. 2020 May 1;11:2128. doi: 10.1038/s41467-020-15989-0 (PMC7195455; doi:10.1038/s41467-020-15989-0)
Supplement: Supplementary file 1 — Supplementary Information [file 41467_2020_15989_MOESM1_ESM.pdf]

Attention amplifies neural representations of changes in sensory input at the expense of perceptual accuracy

Mehrpour et al.

Supplementary Note 1

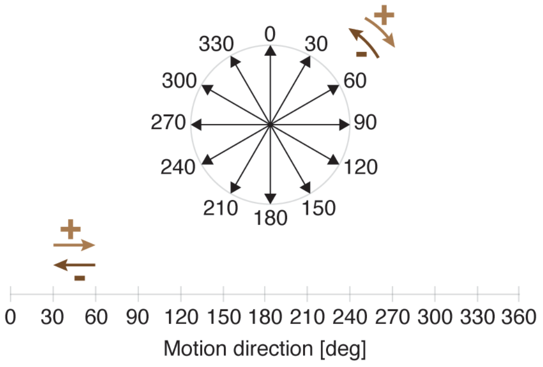

Supplementary Figure 1. Illustration of motion directions and sign convention in both polar (top) and Cartesian (bottom) coordinate systems. Positive and negative signs denote clockwise (rightward) and counterclockwise (leftward) displacements, respectively.

Supplementary Note 2

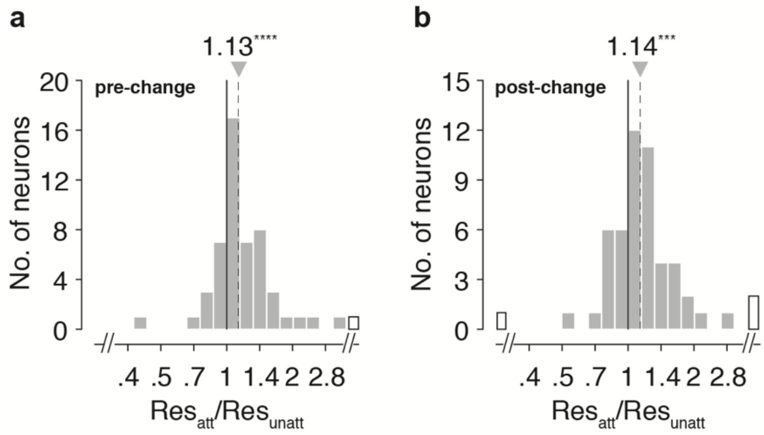

Supplementary Figure 2. Distributions of attentional modulation for a population of MT responses (**a**) 700 ms prior to, and (**b**) 100 – 200 ms following the direction change (n = 52 cells). For each unit we used the median of its attentional modulations across motion directions. Attention significantly enhances MT pre- and post-change neural responses with median values of 13% and 14%, respectively. The position of the medians and the corresponding p values are indicated (vertical dashed lines; *** p = 0.001; **** p = 0.00002 two-sided Wilcoxon signed rank test for distribution with median equal to 1). Unfilled bars represent cells with $response_{att}/response_{unatt}$ less than 0.26 or greater than 3.77**.** Source data are provided as a Source Data file.

Supplementary Note 3

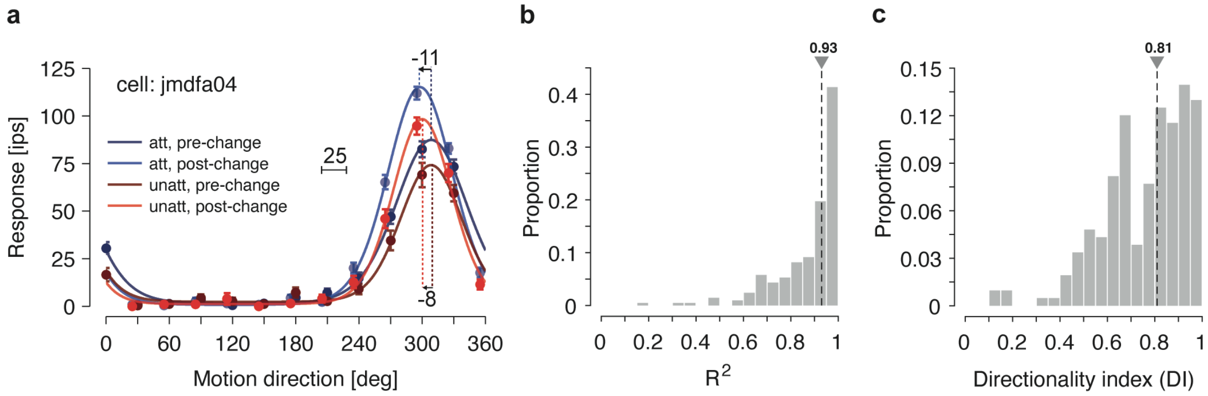

Supplementary Figure 3. Direction tuning curves of MT neurons. (**a**) Pre- and post-change direction tuning curves of an example MT cell in the attended and unattended conditions. Data points depict responses to different directions averaged over either pre- (-700 – 0 ms) or post-change (100 – 200 ms) analysis time windows for either of the two attention conditions. Error bars represent the standard error of the mean. In each condition, we fitted a von Mises function to the cell's responses to 12 directions to estimate the corresponding direction tuning curve (solid lines). Vertical, dotted lines in each condition mark the preferred direction of the cell in that condition. For the direction change of +25°, attention shifted the direction tuning of this cell by -11°. The tuning shift was -8° when the stimulus inside the RF was unattended. (**b**) Distribution of coefficient of determination (R^2) for the von Mises function across cells and conditions (n = 208 = 52 cells x 4 conditions per cell). The median value is marked. (**c**) Distribution of directionality index, DI = (Max Response – Min Response) / (Max Response + Min Response), for the von Mises function across cells and conditions (n = 208). The median value is marked. Source data are provided as a Source Data file.

Supplementary Note 4

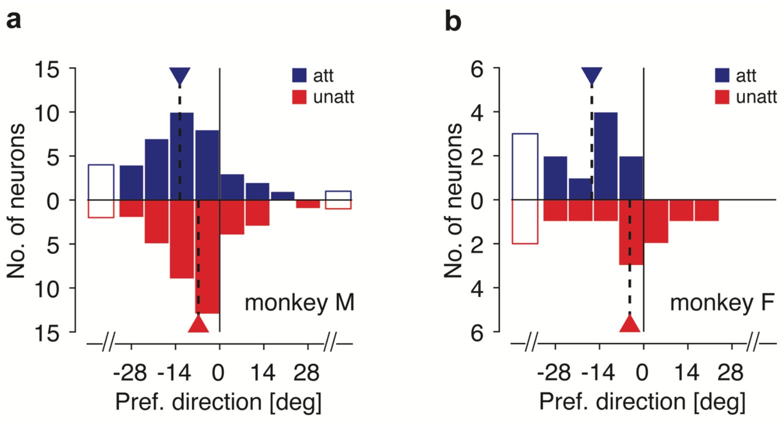

Supplementary Figure 4. Figure 2b replotted for each monkey individually. Distributions of direction tuning curve shifts caused by attended (blue) and unattended (red) direction changes of +25° for (**a**) monkey M: n = 40 cells, and (**b**) monkey F: n = 12 cells. Unfilled bars represent cells for which the magnitude of tuning shift was greater than 40°. The median values (indicated by arrowheads) and the corresponding p values are listed in **Supplementary Table 1**. Source data are provided as a Source Data file.

Monkey	# cells	Attended shift (deg)	Unattended shift (deg)	Attention impact (deg)
**M**	40	-13<br>p = 0.00005	-7<br>p = 0.001	-6<br>p* = 0.03
**F**	12	-17<br>p = 0.0005	-4<br>p = 0.2	-13<br>p* = 0.01

Supplementary Table 1. The median value of direction tuning curve shifts, induced by attended and unattended direction changes of +25° for each monkey individually. p is the p-value of the two-sided Wilcoxon signed rank test for distributions with zero median. p* is the p-value of the paired two-sided Wilcoxon signed rank test for the null hypothesis that the difference between paired samples comes from a distribution with zero median.

Supplementary Note 5

Control analysis 1: We convolved spike trains with a Gaussian kernel to compute the spike density functions. As summarized in **Supplementary Table 2**, the shifts of direction tuning curves in both attended and unattended conditions are independent of the standard deviation used to smooth the spike trains.

σ (ms)	Attended shift (deg)	Unattended shift (deg)	Attention impact (deg)
**10**	-14<br>p = 0.0000005	-7<br>p = 0.0005	-7<br>p* = 0.004
**20**	-14<br>p = 0.0000005	-6<br>p = 0.0005	-8<br>p* = 0.001
**40**	-12<br>p = 0.0000006	-6<br>p = 0.0003	-6<br>p* = 0.01

Supplementary Table 2. The median value of direction tuning curve shifts induced by attended and unattended direction changes of +25° (n = 52 cells). p is the p-value of the two-sided Wilcoxon signed rank test for distributions with zero median. p* is the p-value of the paired two-sided Wilcoxon signed rank test for the null hypothesis that the difference between paired samples comes from a distribution with zero median.

Control analysis 2: To make sure that direction tuning curve shifts do not depend on the tuning characteristics of MT cells, we repeated the analysis by applying the following inclusion criteria: (1) neurons were highly direction selective (response to the preferred direction was at least 5 times larger than the response to the anti-preferred direction), (2) the neurons were well-tuned (goodness of fit > 0.7). 25 out of 52 cells fulfilled these criteria. The results of this analysis (**Supplementary Table 3:** Control analysis 2) were consistent with those presented before (**Fig. 2b**).

Control analysis 3: We performed a control analysis to show that the direction tuning curve shifts are not affected by the selection of the post-change time window. This analysis used an analysis time window from +150 to +250 ms following the direction change to assess the post-change direction tuning curves. The results (**Supplementary Table 3:** Control analysis 3) confirmed those reported in the article (**Fig. 2b**).

Reference	# cells	Attended shift (deg)	Unattended shift (deg)	Attention impact (deg)
**Control analysis 2**	25	-13<br>p = 0.0005	-5<br>p = 0.002	-8<br>p* = 0.02
**Control analysis 3**	52	-10<br>p = 0.0000006	-7<br>p = 0.0001	-3<br>p* = 0.02

Supplementary Table 3. The median value of direction tuning curve shifts induced by attended and unattended direction changes of +25°. p is the p-value of the two-sided Wilcoxon signed rank test for distributions with zero median. p* is the p-value of the paired two-sided Wilcoxon signed rank test for the null hypothesis that the difference between paired samples comes from a distribution with zero median.

Control analysis 4: We demonstrated that the direction tuning curve shift induced by attended and unattended direction changes is independent of the symmetry of the function employed to fit the neural responses to the different directions. We fitted the pre- and post-change data separately with skewed von Mises functions (see Methods). In two separate analyses, we included tuned (goodness of fit greater than 0.7), direction-selective (response to preferred

direction at least 5-fold larger than response to anti-preferred direction) cells based on the fit of the data with each of symmetric (**Supplementary Fig. 5a**) and skewed (**Supplementary Fig. 5b**) von Mises functions each time. The results show that regardless of the symmetry of the function used to model neural responses to different directions, post-change direction tuning curves in attended condition are shifted and the shift is significantly larger than be explained by sensory adaptation in unattended condition.

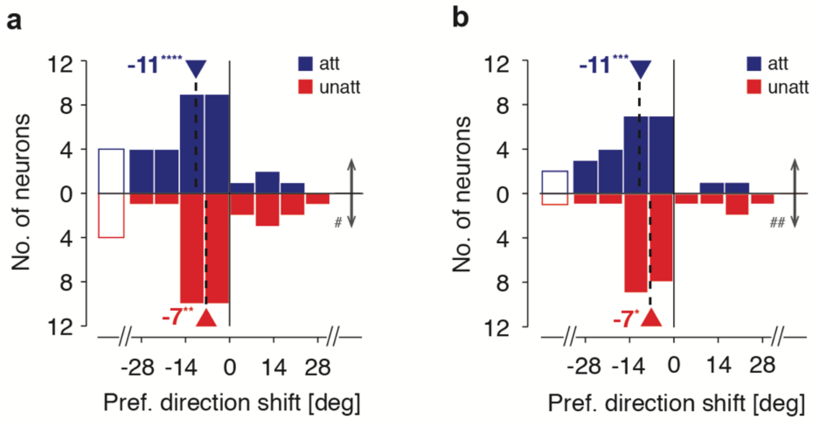

Supplementary Figure 5. Distribution of direction tuning curve shifts caused by attended (blue) and unattended (red) direction changes of +25° across MT cells. The direction tuning curves of each cell were modeled by skewed von Mises functions (see Methods). The median values of the shifts and the corresponding p values are labeled (vertical dashed lines; * $p < 0.05$; ** $p < 0.01$; *** $p < 0.001$; **** $p < 0.0001$ two-sided Wilcoxon signed rank test for distribution with a median of 0; $^{\#}$ $p < 0.05$; $^{\#\#}$ $p < 0.01$ paired two-sided Wilcoxon signed rank test). Unfilled bars represent cells for which the magnitude of tuning shift was greater than 40°. (**a**) Inclusion was based on the results of fitting the data with the skewed von Mises functions: 34 out of 52 neurons fulfilled the inclusion criteria. Tuning shifts were significant in both attended (p = 0.00004, two-sided Wilcoxon signed rank test for distribution with zero median) and unattended (p = 0.009, two-sided Wilcoxon signed rank test for distribution with zero median) conditions. Moreover, the attention-related increase in the tuning shift was statistically significant (p = 0.02, paired two-sided Wilcoxon signed rank test for the null hypothesis that the difference between paired samples comes from a distribution with zero median). (**b**) Inclusion was based on the results of fitting the data with the (symmetric) von Mises functions: 25 out of 52 neurons fulfilled the inclusion criteria. Tuning shift was significant in both attended (p = 0.0005, two-sided Wilcoxon signed rank test for distribution with zero median) and unattended (p = 0.03, two-sided Wilcoxon signed rank test for distribution with zero median) conditions. Moreover, the attention-related increase in tuning shifts was statistically significant (p = 0.009, paired two-sided Wilcoxon signed rank test for the null hypothesis that the difference between paired samples comes from a distribution with zero median). Source data are provided as a Source Data file.

Supplementary Note 6

Supplementary Figure 6 illustrates how the direction tuning shift of single cells induced by an abrupt direction change might result in the overestimation of a perceived direction change in both attended (left column) and unattended (right column) conditions. **Supplementary Figure 6a** plots the tuning curves of four representative neurons (different colors) prior and subsequent to the direction change of +25° with solid and dashed curves, respectively. In each condition, we used the median of the direction tuning parameters across all cells in the corresponding condition to create the tuning curves of the representative cells. This is justified because there was a weak correlation between the pre-change preferred directions of MT cells and the direction tuning shifts induced by the direction change (Pearson r = 0.27, p = 0.053 for attended; and Pearson r = 0.08, p = 0.6 for unattended. Spearman r = 0.19, p = 0.2 for attended; and Spearman r = 0.12, p = 0.4 for unattended). **Supplementary Figure 6b** depicts the population response profiles to a stimulus moving in 25° (solid black curve) and the direction change of 25° (dashed gray curve). Vertical solid black lines show the motion direction, the downward gray triangle and the vertical gray dashed lines indicate the pre- and post-change motion directions, respectively. The population post-change response profiles show peaks shifted away from the post-change direction of +25°. This shift has the same magnitude but opposite sign as the neural direction tuning curve shifts. As single cells exhibit larger tuning shifts in the attended condition than the unattended condition, the post-change response profile in the attended condition is shifted more than that in the unattended condition.

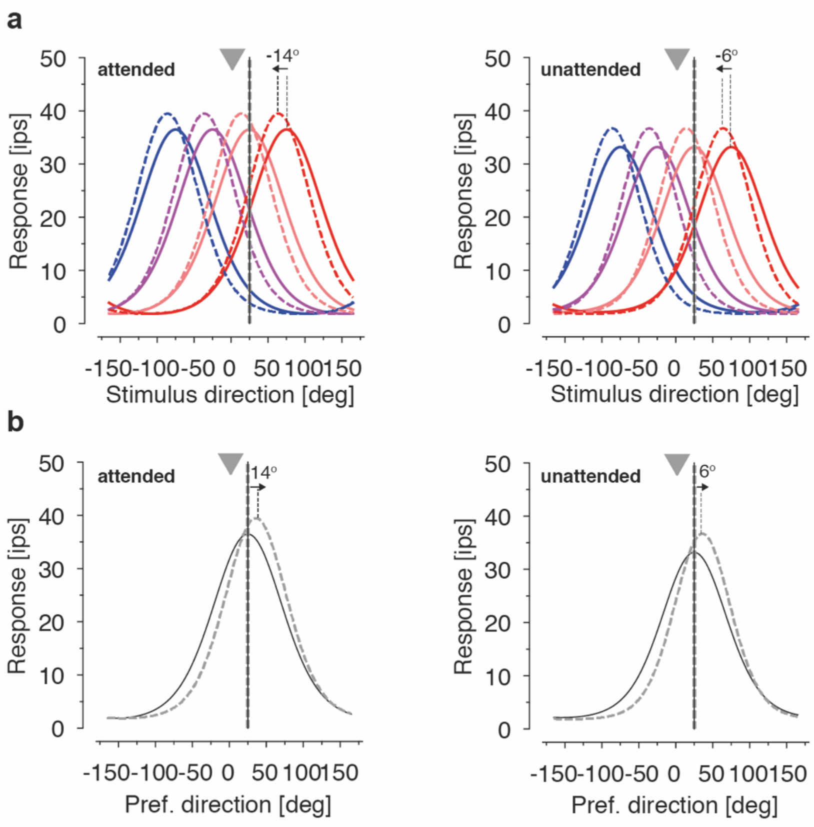

Supplementary Figure 6. Constructing population response profiles using a labeled-line model. (**a**) Direction tuning curves of 4 example neurons (shown in different colors) prior to (solid curves) and following (dashed curves) the direction change of +25° in the attended (left panel) and unattended (right panel) conditions. The black solid line depicts the motion direction, downward gray arrow and gray dashed line mark the stimulus direction prior to and following the direction change, respectively. (**b**) Pre- and post-change population response profiles in attended (left panel) and unattended (right panel) conditions.

Supplementary Note 7

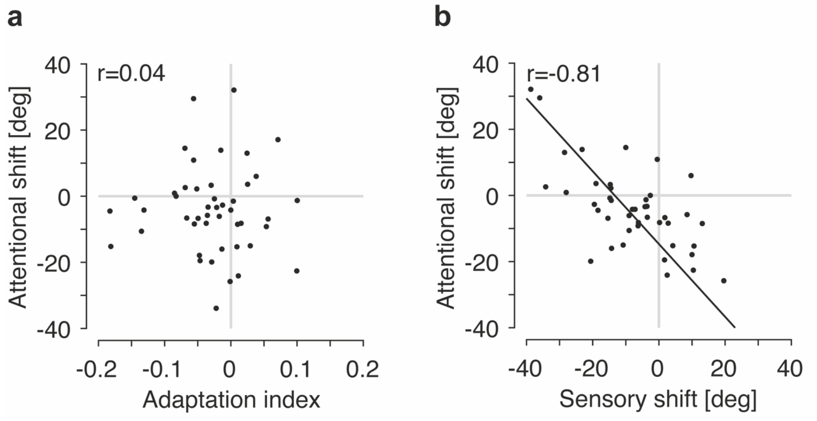

Supplementary Figure 7. Interaction between attention and adaptation in the neural representation of direction changes. (**a**) The attentional shift (i.e. attended shift – unattended shift) is plotted against the visual motion adaptation assessed by the average adaptation index across attentional conditions (n = 52 cells). The adaptation index for each attentional condition is defined as ($R_{pre\text{-}change}$ – $R_{post\text{-}onset}$) / ($R_{pre\text{-}change}$ + $R_{post\text{-}onset}$), where $R_{pre\text{-}change}$ and $R_{\_post\text{-}onset}$ are the cell's average responses *(across directions)* to the pre-change (300 ms prior to the direction change) and post-onset (from 400 to 700 ms) stimuli. Data points corresponding to the attentional shifts with magnitudes greater than 40° are not shown (6 data points). (**b**) Relationship between attentional shift (attended shift – unattended shift), plotted along the y-axis and the sensory (unattended) shift represented along the x-axis (n = 52 cells). Data points corresponding to the shifts with magnitudes greater than 40° are not shown (9 data points). The Pearson correlation coefficients are labeled on each graph. Source data are provided as a Source Data file.
