# Supplementary material for: Attention amplifies neural representations of changes in sensory input at the expense of perceptual accuracy
Source: Nat Commun. 2020 May 1;11:2128. doi: 10.1038/s41467-020-15989-0 (PMC7195455; doi:10.1038/s41467-020-15989-0)
Supplement: Supplementary file 3 — Reporting Summary [file 41467_2020_15989_MOESM3_ESM.pdf]

nature research

Corresponding author(s): Vahid Mehrpour, Stefan Treue

Last updated by author(s): Mar 16, 2020

Reporting Summary

Nature Research wishes to improve the reproducibility of the work that we publish. This form provides structure for consistency and transparency in reporting. For further information on Nature Research policies, see Authors & Referees and the Editorial Policy Checklist.

Statistics

For all statistical analyses, confirm that the following items are present in the figure legend, table legend, main text, or Methods section.

n/a	Confirmed	
☐	☒	The exact sample size (*n*) for each experimental group/condition, given as a discrete number and unit of measurement
☐	☒	A statement on whether measurements were taken from distinct samples or whether the same sample was measured repeatedly
☐	☒	The statistical test(s) used AND whether they are one- or two-sided<br>*Only common tests should be described solely by name; describe more complex techniques in the Methods section.*
☒	☐	A description of all covariates tested
☒	☐	A description of any assumptions or corrections, such as tests of normality and adjustment for multiple comparisons
☐	☒	A full description of the statistical parameters including central tendency (e.g. means) or other basic estimates (e.g. regression coefficient) AND variation (e.g. standard deviation) or associated estimates of uncertainty (e.g. confidence intervals)
☐	☒	For null hypothesis testing, the test statistic (e.g. *F*, *t*, *r*) with confidence intervals, effect sizes, degrees of freedom and *P* value noted<br>*Give P values as exact values whenever suitable.*
☒	☐	For Bayesian analysis, information on the choice of priors and Markov chain Monte Carlo settings
☒	☐	For hierarchical and complex designs, identification of the appropriate level for tests and full reporting of outcomes
☐	☒	Estimates of effect sizes (e.g. Cohen's *d*, Pearson's *r*), indicating how they were calculated

Our web collection on statistics for biologists contains articles on many of the points above.

Software and code

Policy information about availability of computer code

Data collection	Monkey electrophysiology: A computer program running on an Apple Macintosh PowerPC controlled the stimulus presentations and recorded the behavior of the animals.<br><br>Human psychophysics: Stimuli were programmed and controlled in an open-source software package MWorks version 0.5 (http://mworks-project.org/) running on a Macintosh computer.<br><br>The analysis code used in this study is available from the corresponding authors upon reasonable request.
Data analysis	All data analysis was performed using MATLAB R2015a (MathWorks, Natick, MA).<br><br>The analysis code used in this study is available from the corresponding authors upon reasonable request.

For manuscripts utilizing custom algorithms or software that are central to the research but not yet described in published literature, software must be made available to editors/reviewers. We strongly encourage code deposition in a community repository (e.g. GitHub). See the Nature Research guidelines for submitting code & software for further information.

Data

Policy information about availability of data

All manuscripts must include a data availability statement. This statement should provide the following information, where applicable:

- Accession codes, unique identifiers, or web links for publicly available datasets
- A list of figures that have associated raw data
- A description of any restrictions on data availability

The source data underlying all figures and supplementary figures are provided as a 'Source Data' file. Any additional data from this study are available from the corresponding authors upon reasonable request. A reporting summary for this Article is available as a Supplementary Information file.

Field-specific reporting

Please select the one below that is the best fit for your research. If you are not sure, read the appropriate sections before making your selection.

☒ Life sciences ☐ Behavioural & social sciences ☐ Ecological, evolutionary & environmental sciences

For a reference copy of the document with all sections, see nature.com/documents/nr-reporting-summary-flat.pdf

Life sciences study design

All studies must disclose on these points even when the disclosure is negative.

Sample size	Monkey electrophysiology: Per the standard of the field, recordings were performed in two monkeys (40 neurons from monkey M and 12 from monkey F) to ensure our findings were consistent across both animals.<br><br>Human psychophysics: We recruited n=10 human subjects in our human study to ensure our behavioral results were consistent across the subjects. This number also gave us the statistical power to draw conclusions about the population of human subjects.
Data exclusions	Monkey electrophysiology: No data exclusion.<br><br>Human Psychophysics: Of 21 participants, 10 subjects fulfilled the criterion for inclusion in this study: sensitivity in discriminating between upward and downward motion in a direction discrimination experiment (an average discrimination threshold less than 3 deg for both rightward and leftward motion).
Replication	Monkey electrophysiology: Our results were successfully replicated across two monkeys.<br><br>Human psychophysics: Our results were successfully replicated across all but one of the subjects.
Randomization	Monkey electrophysiology: direction change times were sampled from a bimodal probability distribution throughout the recording sessions.<br>Human psychophysics: In each trial, the direction of motion was randomly (uniform distribution) chosen to be leftward or rightward. A clockwise (CW) or counterclockwise (CCW) direction change of 22deg, 25deg, or 27deg occurred in the direction of right RDP at a random time (uniform distribution). Both the direction change (CW or CCW) and its magnitude were randomized (uniform distributions) in each trial.
Blinding	In both data collection and data analysis, there was no room for human biases as all steps were random (e.g. selection of samples including monkeys, MT units, and human subjects) and all procedures were automatized.

Reporting for specific materials, systems and methods

We require information from authors about some types of materials, experimental systems and methods used in many studies. Here, indicate whether each material, system or method listed is relevant to your study. If you are not sure if a list item applies to your research, read the appropriate section before selecting a response.

Materials & experimental systems

n/a	Involved in the study	
☒	☐	Antibodies
☒	☐	Eukaryotic cell lines
☒	☐	Palaeontology
☐	☒	Animals and other organisms
☐	☒	Human research participants
☒	☐	Clinical data

Methods

n/a	Involved in the study	
☒	☐	ChIP-seq
☒	☐	Flow cytometry
☒	☐	MRI-based neuroimaging

Animals and other organisms

Policy information about studies involving animals; ARRIVE guidelines recommended for reporting animal research

Laboratory animals	Data were collected from two male rhesus macaques. The animals were five to seven years old.
Wild animals	No wild animals were used in the study.
Field-collected samples	No field collected samples were used in the study.
Ethics oversight	Animal procedures complied with all applicable German and European regulations, as well as the NIH Guide for Care and Use of Laboratory Animals and were approved by the responsible regional government office.

Note that full information on the approval of the study protocol must also be provided in the manuscript.

Human research participants

Policy information about studies involving human research participants

Population characteristics	Twenty-one volunteers, 7 males and 14 females, aged 21-35, with normal or corrected-to-normal vision took part in the experiment.
Recruitment	Participants were students/researchers at the University of Goettingen and had no prior knowledge about the current study. We used fliers and the database of previous participants to invite subjects to participate in our study. We did not have any bias in the process of selecting subjects.
Ethics oversight	The study was approved by the Ethics Committee of the Georg-Elias-Mueller Institute of Psychology of the Faculty for Biology and Psychology, University of Goettingen, and all participants signed a written consent form prior to the experiment.

Note that full information on the approval of the study protocol must also be provided in the manuscript.
