# Supplementary material for: Attention amplifies neural representations of changes in sensory input at the expense of perceptual accuracy
Source: Nat Commun. 2020 May 1;11:2128. doi: 10.1038/s41467-020-15989-0 (PMC7195455; doi:10.1038/s41467-020-15989-0)
Supplement: Supplementary file 4 — Source Data [file 41467_2020_15989_MOESM4_ESM.zip › Source Data/ReadMe.rtf]

ReadMe for Source Data———————————————————————————————————————data_Fig1_bc.mat is a MATLAB file containing the data used to plot Figure 1b-c.(*) 'timeAxis_Data’ is a structure containing the data used to plot the x axis of the color code plot in attended (timeAxis_Data.att) and unattended (timeAxis_Data.unatt) conditions.(*) 'dirAxis_Data’ is a structure containing the data used to plot the y axis of the color code plot in attended (dirAxis_Data.att) and unattended (dirAxis_Data.unatt) conditions.(*) 'popResp_Data’ is a structure containing the data used to plot the z axis of the color code plot in attended (popResp_Data.att) and unattended (popResp_Data.unatt) conditions.———————————————————————————————————————data_Fig1_de.mat is a MATLAB file containing the data used to plot Figure 1d-e.(*) 'timeAxis_Data’ is a structure containing the data used to plot the x axis of the color code plot in attended (timeAxis_Data.att) and unattended (timeAxis_Data.unatt) conditions.(*) 'dirAxis_Data’ is a structure containing the data used to plot the y axis of the color code plot in attended (dirAxis_Data.att) and unattended (dirAxis_Data.unatt) conditions.(*) 'popResp_Data’ is a structure containing the data used to plot the z axis of the color code plot in attended (popResp_Data.att) and unattended (popResp_Data.unatt) conditions.(*) 'timeAxisTC_Data' is a structure containing the data used to plot the x axis of the black curve in attended (timeAxisTC_Data.att) and unattended (timeAxisTC_Data.unatt) conditions.(*) 'prefDirTC_Data' is a structure containing the data used to plot the y axis of the black curve in attended (prefDirTC_Data.att) and unattended (prefDirTC_Data.unatt) conditions.———————————————————————————————————————data_Fig2_a.mat is a MATLAB file containing the data used to plot Figure 2a.(*) 'dirAxis_Data'  is a structure containing the data used to plot the x axis of the population response curves in each condition:'dirAxis_Data.att.preCh’ corresponds to attended, pre-change condition'dirAxis_Data.att.postCh’ corresponds to attended, post-change condition 'dirAxis_Data.unatt.preCh’ corresponds to unattended, pre-change condition 'dirAxis_Data.unatt.postCh’ corresponds to unattended, post-change condition (*) 'dirAxis_Data'  is a structure containing the data used to plot the x axis of the population response curves in each condition:'dirAxis_Data.att.preCh’ corresponds to attended, pre-change condition'dirAxis_Data.att.postCh’ corresponds to attended, post-change condition 'dirAxis_Data.unatt.preCh’ corresponds to unattended, pre-change condition 'dirAxis_Data.unatt.postCh’ corresponds to unattended, post-change condition———————————————————————————————————————data_Fig2_b.mat is a MATLAB file containing the data used to plot Figure 2b.(*) 'prefDirShift’ is a structure containing the data of direction tuning shifts in attended (prefDirShift.att) and unattended (prefDirShift.unatt) conditions.———————————————————————————————————————data_Fig3_c.mat is a MATLAB file containing the data used to plot Figure 3c.(*) 'subjectNames’ is a cell array containing the name of subjects(*) 'data_perceptionError' is a matrix of #rows = 2 x number of sessions (2 because each session includes rightward and leftward directions) by #columns = number of subjects + 1 containing all the data points for error in the perceived direction change for the subjects listed in 'subjectNames'(*) 'median_perceptionError' is a vector containing average error in the perceived direction change for the subjects listed in 'subjectNames'. To compute the median, we first averaged the errors across rightward and leftward directions in each session and then computed median error across sessions.(*) 'error_perceptionError’ is a vector containing the data of the error bars. To compute the MAD, we first averaged the errors across rightward and leftward directions in each session and then computed MAD across sessions.———————————————————————————————————————data_FigSI2.mat is a MATLAB file containing the data used to plot Figure SI2a-b.(*) 'responseRatio’ is a structure containing the data of ratio of responses in attended to unattended condition prior to (Fig SI2a) and following (Fig SI2b) the direction change(*) 'responseRatio.preChange' is a vector containing data before the direction change(*) 'esponseRatio.postChange’ is a vector containing data after the direction change———————————————————————————————————————data_FigSI3_b.mat is a MATLAB file containing the data used to plot Figure SI3b.(*) 'dirData'  is a structure containing the motion directions in each condition:'dirData.att.preChange’ corresponds to attended, pre-change condition'dirData.att.postChange’ corresponds to attended, post-change condition 'dirData.unatt.preChange’ corresponds to unattended, pre-change condition 'dirData.unatt.postChange’ corresponds to unattended, post-change condition (*) 'firingRateData'  is a structure containing the mean firing rate data in each condition:'firingRateData.att.preChange’ corresponds to attended, pre-change condition'firingRateData.att.postChange’ corresponds to attended, post-change condition 'firingRateData.unatt.preChange’ corresponds to unattended, pre-change condition 'firingRateData.unatt.postChange’ corresponds to unattended, post-change condition(*) 'SEM_firingRateData'  is a structure containing the SEM of firing rate data in each condition:'SEM_firingRateData.att.preChange’ corresponds to attended, pre-change condition'SEM_firingRateData.att.postChange’ corresponds to attended, post-change condition 'SEM_firingRateData.unatt.preChange’ corresponds to unattended, pre-change condition 'SEM_firingRateData.unatt.postChange’ corresponds to unattended, post-change condition———————————————————————————————————————data_FigSI3_c.mat is a MATLAB file containing the data used to plot Figure SI3c.(*) 'rSquared_Data'  is a vector containing the data of R^2 distribution———————————————————————————————————————data_FigSI3_d.mat is a MATLAB file containing the data used to plot Figure SI3d.(*) 'DI_Data'  is a vector containing the data of directionality index———————————————————————————————————————data_FigSI4_a.mat is a MATLAB file containing the data used to plot Figure SI4a.(*) 'prefDirShift’ is a structure containing the data of direction tuning shifts in attended (prefDirShift.att) and unattended (prefDirShift.unatt) conditions.———————————————————————————————————————data_FigSI4_b.mat is a MATLAB file containing the data used to plot Figure SI4b.(*) 'prefDirShift’ is a structure containing the data of direction tuning shifts in attended (prefDirShift.att) and unattended (prefDirShift.unatt) conditions.———————————————————————————————————————data_FigSI5_a.mat is a MATLAB file containing the data used to plot Figure SI5a.(*) 'prefDirShift’ is a structure containing the data of direction tuning shifts in attended (prefDirShift.att) and unattended (prefDirShift.unatt) conditions.———————————————————————————————————————data_FigSI5_b.mat is a MATLAB file containing the data used to plot Figure SI5b.(*) 'prefDirShift’ is a structure containing the data of direction tuning shifts in attended (prefDirShift.att) and unattended (prefDirShift.unatt) conditions.———————————————————————————————————————data_FigSI7_a.mat is a MATLAB file containing the data used to plot Figure SI7a.(*) 'adaptationIndex’ is a vector containing adaptation index data for the population (*) 'attentionalShift’ is a vector containing attentional shift data———————————————————————————————————————data_FigSI7_b.mat is a MATLAB file containing the data used to plot Figure SI7b.(*) 'unattendedShift’ is a vector containing the direction tuning shift data for the population in unattended condition(*) 'attentionalShift’ is a vector containing attentional shift data
